# Human papillomavirus vaccine knowledge and conspiracy beliefs among secondary school students in Lebanon

**DOI:** 10.1186/s12887-023-04177-w

**Published:** 2023-07-15

**Authors:** Joe Khalil, Sarah Boutros, Abdo Hassoun, Souheil Hallit, Habib Barakat

**Affiliations:** 1 School of Medicine and Medical Sciences, Holy Spirit University of Kaslik, P.O. Box 446, Jounieh, Lebanon; 2 Applied Science Research Center, Applied Science Private University, Amman, Jordan; 3 Research Department, Psychiatric Hospital of the Cross, Jal Eddib, Lebanon; 4 Obstetrics and Gynecology Department, Notre Dame, Secours University Hospital Center, Street 93, 3 Byblos, Lebanon

**Keywords:** HPV, HPV awareness, HPV vaccine, Vaccine conspiracy beliefs, Secondary school students, Adolescents

## Abstract

**Background:**

Human Papillomavirus (HPV) is widely prevalent across the globe. In Lebanon, the society is transitioning from traditional conservatism to a more open attitude. Although previous studies have examined the knowledge of adults in Lebanon with regard to HPV and its vaccine, there is a lack of research on secondary school students. Moreover, HPV is considered a worldwide public health matter that needs to be addressed. Therefore, the objective of our study is to assess factors associated with knowledge and conspiracy beliefs towards HPV vaccine among a sample of Lebanese adolescents.

**Methods:**

Between December 2022 and February 2023, we conducted a cross-sectional study on Lebanese adolescents aged 15 to 18 years old. Parental approval was needed in order to participate. We used a questionnaire to collect data, which included the HPV-knowledge questionnaire (HPV-KQ) and the Vaccine Conspiracy Belief Scale (VCBS).

**Results:**

Of the 406 participants who filled the survey, 64.8% were female, with a mean age of 16.62 ± 1.01. Results showed that 31.0% of students had high knowledge about HPV, while 27.6% had high conspiracy beliefs, and 48% of participants relied on the internet to access information on HPV. Students who had previously heard of HPV, received sexual education at school or outside, and had received at least one dose of the HPV vaccine demonstrated significantly higher knowledge of HPV. Additionally, students with high knowledge had a lower mean House Crowding Index, and those whose fathers had a university education had lower conspiracy beliefs. Females had a higher vaccination rate than males, while no significant difference was found between those who had engaged in sexual activity and those who had not. The multivariate analysis indicated that previous awareness of HPV and receiving sexual education outside school were significantly associated with higher knowledge.

**Conclusion:**

Our study brings to light the urgent need for action to increase HPV awareness and vaccination among Lebanese secondary school students. The prevalence of vaccine misconceptions and conspiracy beliefs and the limited knowledge of HPV underscore the importance of more comprehensive sexual education in schools and the dissemination of accurate information about HPV and its vaccine. Furthermore, given the low vaccination rate among males, efforts should be made to promote HPV vaccination among this population. Addressing these issues can improve public health and help prevent the spread of HPV and its related diseases.

## Introduction

Human papillomavirus (HPV) is very prevalent in the population worldwide [1]. The Centers for Disease Control and Prevention (CDC) predicts that over a lifetime, approximately every person will contract the virus [1]. HPV is transmitted through direct skin-to-skin contact, hence it is considered of being one of the many known sexually transmitted infections (STIs; HIV, syphilis, gonorrhea, chlamydia). It can be contracted from having oral, vaginal, or anal sex, even if the person affected was asymptomatic [2]. One prospective study found that HPV infection might even happen between two to three months following the first sexual intercourse in a previously negative woman [3]. Though the virus mostly manifests through benign lesions such as skin condylomas and warts, it can sometimes lead to cancer [4], yet spontaneous clearing of the virus usually happens within the following months after the contraction of the pathogen, with approximately 90% resolution within 2 years [4].

Approximately 200 strands of HPV have been identified [5]. Strands 6 and 11 cause benign anogenital warts, whereas 70% of cervical cancers and other anogenital cancers are related to the high-risk strands 16 and 18 [6], for they cause a chronic infection with the inability of the virus to be cleared by the immune system [4]. High-risk HPV types 31, 33, 45, 52, and 58 cause an additional 15% of cervical malignancies [6]. Moreover, HPV has also been found to cause almost 70% of penile and oropharyngeal caners [7].

There are currently 3 vaccines available on the market; the bivalent vaccine “Cervarix”, which covers the 16–18 HPV strands, the quadrivalent vaccine “Gardasil”, which covers the 6-11-16-18 strands [8], and the most recent one “Gardasil 9”, a 9 valent vaccine that protects against the 6, 11, 16, 18, 31, 33, 45, 52, and 58 strands [9]. The World Health Organization recommends one dose of HPV vaccine to offer protection against cervical cancer [10]. In addition, the “Gardasil 9” vaccine has also received the FDA approval for its usage in both men and women aged from 26 to 45 years [11]. The two doses regimen are given at an interval of 6 to 12 months between each shot, whereas the three doses regimen is given at 0, 1–2 months, 6 months interval for each shot of the vaccine respectively [10].

It is estimated that 2.2% of women in Lebanon’s general population currently have cervical HPV infection [12]. In a cohort group of 1,026 Lebanese women between the ages of 18 and 76 years who were seeking routine gynecological care at a tertiary care facility, the prevalence of HPV reached 4.9% [13]. Moreover, there is currently no government-backed vaccination program in Lebanon, and third-party payers do not cover the expenses of HPV vaccination [14]. Individual decision must be taken on whether the person wants to get the HPV vaccine or not [12]. According to the Lebanese Ministry of Public Health, the “Gardasil 9” vaccine is priced at 180 USD, and the “Cervarix” is priced at 70.95 USD [15].

Traditional perceptions of Lebanon place the community as a conservative society with a low incidence of STIs, however, today’s youth are less rigidly bound by their social norms than they were in the past [14]. Cervical cancer has been placed as the sixth most common cancer in Lebanon among girls and women aged between 14 and 44 [16]. Moreover, a systematic review done in 2021 showed that Lebanon ranked first among 26 different countries, with an 86% occurrence of HPV-positive cases, with HPV-16 being the most predominant subtype [17]. On the other hand, a previous study conducted on Lebanese physicians of different specialties (Obstetrics and Gynecology, Family Medicine, Pediatrics, and Infectious Diseases) showed that Lebanese physicians, do not follow the international guidelines in recommending vaccination against HPV. Females were 6.8 times more likely to be offered the vaccine compared to males and were less likely to be recommended the vaccine if they said they were not sexually active or if minors presented to the physician without their parents [18]. In Lebanon, vaccination rates among female schoolgirls and university students were 2.5% [12] and 16.5% [14] respectively.

Higher conspiracy beliefs have also been demonstrated to be associated with higher refusal of getting the HPV vaccine in Canada [19] and in Jordanian female university students [20]. Furthermore, a nationwide study conducted in Brazil during 2018 on 8581 participants, emphasized on the importance of implementing educational initiatives on HPV within the community in order to enhance the overall vaccination rates [21]. Another study conducted on high school students in China, exposed the lack of awareness of students to HPV infection with no intention to receiving the vaccine [22]. Yet, a study done in Italy showed that high school students intended to receive the HPV vaccine regardless of their knowledge about HPV [23]. Additionally, a study conducted in 2020 in different Arab countries showed that knowledge about HPV infection and vaccination was insufficient, and that awareness is necessary among Arab communities [24]. Studies conducted in Lebanon about HPV awareness and vaccination rate were very few and limited to either parents [12] or university students [14–25]. To our knowledge, no studies have been previously conducted directly on the minor Lebanese population, with no sufficient international data covering this topic. Therefore, the objective of our study was to assess factors associated with knowledge and conspiracy beliefs towards HPV vaccine among a sample of Lebanese adolescents.

## Methods

### Study Design and Sampling

A cross-sectional study was conducted from December 2022 till February 2023. Data was collected by sharing the questionnaire through social media applications using the snowball technique and spreading the questionnaire to different adolescents. Consequently, those who enrolled in the study were asked to share the questionnaire with their friends and family members they knew from the same age category. Inclusion criteria were Lebanese males and females secondary school student from grade 10, 11, or 12 between the ages of 15 to 18. A total of 412 students accepted to fill the questionnaire, after getting parental approval. Among those 412, 6 students were then excluded because they were under 15 years old. Hence, 406 students were included in the final database.

### Minimal sample size calculation

Minimal sample size calculation was done using the G-power software version 3.0.10, which came out to be 395, by using the linear multiple regression test, and using an effect size f^2^ = 0.02, alpha error of 0.05, power = 0.8, and 13 predictors to be used in the analysis.

### Questionnaire

The questionnaire was done in the English language, and included socio-demographic data (i.e. age, sex, parental income, House Crowding Index (number of person/room in the house) [26], their vaccination status, the HPV knowledge questionnaire (HPV-KQ) [27], and vaccination conspiracy beliefs scale [19].

#### Human papillomavirus knowledge questionnaire

The Human Papillomavirus Knowledge Questionnaire consists of 13 questions that inquire about general information on HPV transmission, symptoms, and vaccination. Students were required to respond to each question by selecting one of three options: “True”, “False”, or “I don’t know”. A score of 1 point was assigned for each correct answer, while incorrect and “I don’t know” answers were given a score of 0 [27]. Higher scores reflect higher knowledge.

#### Vaccination conspiracy beliefs scale

The vaccination conspiracy beliefs scale (VCBS) is made of seven statements where students expressed their level of agreement or disagreement with a particular statement using a 7-point Likert scale that ranges from “strongly disagree” to “strongly agree” [19]. Higher scores indicate higher conspiracy beliefs [28].

### Statistical analysis

Statistical analysis was performed using SPSS software version 22. Reliability analysis was evaluated using Cronbach’s alpha values. The 75th percentile was used as a cutoff point to divide participants into low/high knowledge and conspiracy beliefs [29]. To compare two categories, the Chi-square/Fisher test was used, while the Student t-test was used to compare two means. Two logistic regressions were conducted afterwards, taking the dichotomized knowledge and conspiracy beliefs scores as the dependent variables respectively. Factors that showed a *p* < 0.25 in the bivariate analysis were entered as independent variables in the regressions. *P* < 0.05 was considered statistically significant.

## Results

**Fig. 1:**
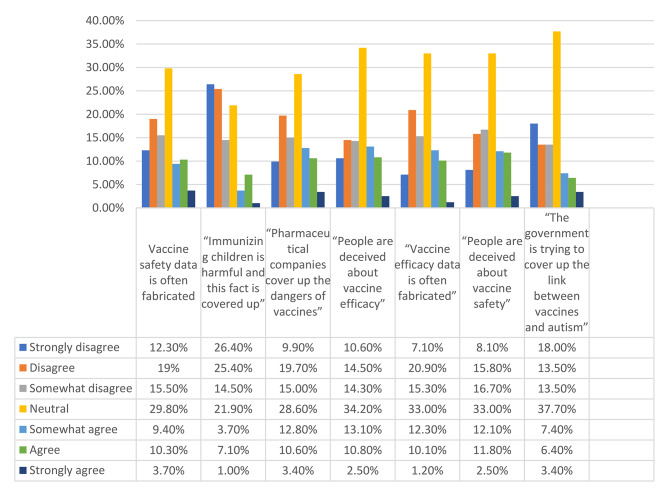
Description of the answers of the conspiracy beliefs items

The Cronbach’s alpha values were good for the knowledge and conspiracy beliefs scales (α = 0.89 for both). The mean age was 16.62 ± 1.01 years (min = 15; max = 18), with 64.8% females. Moreover, 126 (31.0%) of the students had high knowledge (75th percentile cutoff = 7), and 112 (27.6%) showed high conspiracy beliefs (75th percentile cutoff = 29). All details of the participants are summarized in Table 1. The description of the answers to the items of the knowledge and conspiracy belief scales is found in Table 2; Fig. 1 respectively.

**Table 1:** Sociodemographic and other characteristics of the participants (n = 406)

	n (%)
**Gender**	
Male	143 (35.2%)
Female	263 (64.8%)
**Religion**	
Christian	272 (67.0%)
Muslim	126 (31.0%)
Other	8 (2.0%)
**Governorate**	
Beirut	88 (21.7%)
Mount Lebanon	270 (66.5%)
North	20 (4.9%)
South	15 (3.7%)
Bekaa	13 (3.2%)
**Paternal level of education**	
Primary	39 (9.6%)
Complementary	46 (11.3%)
Secondary	133 (32.8%)
University	188 (46.3%)
**Maternal level of education**	
Primary	28 (6.9%)
Complementary	31 (7.6%)
Secondary	121 (29.8%)
University	226 (55.7%)
**Sexual life**	
Never had a sexual activity	336 (82.8%)
Had prior sexual activity	30 (7.4%)
Currently sexually active	40 (9.9%)
**Ever heard of HPV**	
No	217 (53.4%)
Yes	189 (46.6%)
**Heard about HPV from**:	
Parents	68 (16.7%)
Doctor	89 (21.9%)
School	128 (31.5%)
Partner	24 (5.9%)
Friends	85 (20.9%)
Internet	195 (48.0%)
Television	77 (19.0%)
**Ever received sexual education**	
No	130 (32.0%)
Yes, at school	136 (33.5%)
Yes, other than school	140 (34.5%)
**Have received at least one dose of HPV vaccine**	
No	212 (52.2%)
Yes	46 (11.3%)
I do not know	148 (36.5%)
	**Mean ± SD**
**Age (in years)**	16.62 ± 1.01
**Household crowding index (person/room)**	1.04 ± 0.54

**Table 2:** Description of the answers of the knowledge items

	Right answer
“Only women can get infected with HPV”	161 (39.7%)
“HPV can cause cervical cancer in women”	171 (42.1%)
“HPV can cause cancer in areas such as the head and neck”	68 (16.7%)
“HPV causes cancer in women only”	132 (32.5%)
“HPV can cause genital warts”	145 (35.7%)
“A person could have HPV for many years without knowing it”	173 (42.6%)
“HPV is transmitted through sex”	204 (50.2%)
“Most people infected with HPV have visible signs or symptoms of the infection”	85 (20.9%)
“A person’s chances of getting HPV increase with the number of sexual partners they have”	160 (39.4%)
“Nearly all sexually active people will contract HPV at some point”	61 (15.0%)
“The HPV vaccine is only recommended for girls”	114 (28.1%)
“Full protection against HPV requires more than 1 dose of the vaccine”	93 (22.9%)
“The HPV vaccine is most effective if given to people who have not yet started having sex”	107 (26.4%)

### Bivariate analysis

A higher percentage of students who have previously heard of HPV, those who have received sexual education at school or outside it, and those who have received at least one dose of the HPV vaccine had a significantly high level of knowledge of HPV. Moreover, a lower mean HCI was significantly found in students with high knowledge. Furthermore, a lower percentage of students whose fathers have a university level of education, had high conspiracy beliefs (Table 3).

**Table 3:** Bivariate analysis of factors associated with low/high knowledge and conspiracy belief

	Knowledge			Conspiracy belief		
	Low	High	*p*	Low	High	*p*
**Gender**			0.097			0.423
Male	106 (74.1%)	37 (25.9%)		107 (74.8%)	36 (25.2%)	
Female	174 (66.2%)	89 (33.8%)		187 (71.1%)	76 (28.9%)	
**Region of living**			0.485			0.102
Beirut/ Mount Lebanon	249 (69.6%)	109 (30.4%)		264 (73.7%)	94 (26.3%)	
North/ South/ Bekaa	31 (64.6%)	17 (35.4%)		30 (62.5%)	18 (37.5%)	
**Religion**			0.052			0.388
Christian	183 (67.3%)	89 (32.7%)		201 (73.9%)	71 (26.1%)	
Muslim	94 (74.6%)	32 (25.4%)		86 (68.3%)	40 (31.7%)	
Other	3 (37.5%)	5 (62.5%)		7 (87.5%)	1 (12.5%)	
**Paternal level of education**			0.166			**0.038**
Primary	29 (74.4%)	10 (25.6%)		25 (64.1%)	14 (35.9%)	
Complementary	36 (78.3%)	10 (21.7%)		30 (65.2%)	16 (34.8%)	
Secondary	95 (71.4%)	38 (28.6%)		90 (67.7%)	43 (32.3%)	
University	120 (63.8%)	68 (36.2%)		149 (79.3%)	39 (20.7%)	
**Maternal level of education**			0.556			0.087
Primary	22 (78.6%)	6 (21.4%)		18 (64.3%)	10 (35.7%)	
Complementary	23 (74.2%)	8 (25.8%)		20 (64.5%)	11 (35.5%)	
Secondary	80 (66.1%)	41 (33.9%)		81 (66.9%)	40 (33.1%)	
University	155 (68.6%)	71 (31.4%)		175 (77.4%)	51 (22.6%)	
**Sexual life**			0.311			0.312
Never had a sexual activity	237 (70.5%)	99 (29.5%)		239 (71.1%)	97 (28.9%)	
Had prior sexual activity	19 (63.3%)	11 (36.7%)		22 (73.3%)	8 (26.7%)	
Currently sexually active	24 (60.0%)	16 (40.0%)		33 (82.5%)	7 (17.5%)	
**Ever heard of HPV**			**< 0.001**			0.800
No	186 (85.7%)	31 (14.3%)		156 (71.9%)	61 (28.1%)	
Yes	94 (49.7%)	95 (50.3%)		138 (73.0%)	51 (27.0%)	
**Ever received sexual education**			**0.001**			0.340
No	106 (81.5%)	24 (18.5%)		89 (68.5%)	41 (31.5%)	
Yes, at school	87 (64.0%)	49 (36.0%)		98 (72.1%)	38 (27.9%)	
Yes, other than school	87 (62.1%)	53 (37.9%)		107 (76.4%)	33 (23.6%)	
**Have received at least one dose of HPV vaccine**			**< 0.001**			0.252
No	156 (73.6%)	56 (26.4%)		147 (69.3%)	65 (30.7%)	
Yes	14 (30.4%)	32 (69.6%)		37 (80.4%)	9 (19.6%)	
I don’t know	110 (74.3%)	38 (25.7%)		110 (74.3%)	38 (25.7%)	
**Knowledge**						0.309
Low				207 (73.9%)	73 (26.1%)	
High				87 (69.0%)	39 (31.0%)	
**Age**	16.68 ± 0.99	16.50 ± 1.05	0.107	16.61 ± 1.00	16.65 ± 1.05	0.703
**Household crowding index**	1.08 ± 0.58	0.94 ± 0.41	**0.005**	1.04 ± 0.55	1.03 ± 0.50	0.945

Numbers in bold indicate significant *p* values

### Vaccination rate between genders and sexual activity

A higher vaccination rate was significantly found in females compared to males (n = 40/263; 15.2% vs. 6/143; 4.6%). No significant difference in vaccination rate was found between those who have ever had a sexual activity compared to not (n = 13/70; 28.3% vs. n = 34/70; 16.0%).

### Multivariable analysis

Having heard of the HPV (aOR = 6.06) and having received sexual education outside the school compared to not (aOR = 2.01) were significantly associated with high knowledge (Table 4, Model 1). None of the variables was significantly associated with high conspiracy beliefs (Table 4, Model 2).

**Table 4:** Multivariable analyses

	*p*	aOR	95% CI
Model 1: Logistic regression taking high vs. low* knowledge as the dependent variable (R^**2**^ **= 0.266)**			
Gender (females vs. males*)	0.412	1.24	0.74; 2.09
Religion	0.417		
Christian		1	
Muslim	0.729	0.90	0.49; 1.64
Other	0.221	2.66	0.55; 12.80
Paternal level of education	0.185		
Primary		1	
Complementary	0.127	0.41	0.13; 1.29
Secondary	0.093	0.44	0.17; 1.15
University	0.412	0.68	0.27; 1.71
Ever heard about HPV (yes vs. no*)	**< 0.001**	6.06	3.63; 10.11
Ever received sexual education (yes vs. no*)	0.080		
No		1	
Yes, at school	0.077	1.76	0.94; 3.28
Yes, other than school	**0.029**	2.01	1.07; 3.78
Age	0.057	0.78	0.61; 1.01
Household crowding index	0.142	0.67	0.39; 1.15
Model 2: Logistic regression taking high vs. low* conspiracy beliefs as the dependent variable (R^**2**^ **= 0.044)**			
Paternal level of education	0.250		
Primary		1	
Complementary	0.978	1.01	0.39; 2.63
Secondary	0.732	0.87	0.39; 1.92
University	0.156	0.54	0.23; 1.26
Maternal level of education	0.753		
Primary		1	
Complementary	0.950	1.04	0.33; 3.24
Secondary	0.923	1.05	0.42; 2.62
University	0.613	0.78	0.30; 2.02
Region of living (North/South/Bekaa vs. Beirut/Mount Lebanon*)	0.105	1.70	0.89; 3.25

*Reference group; numbers in bold indicate significant *p* values

## Discussion

To our knowledge, and despite the prevalence of HPV infections, there is no prior research that has focused on the information and attitudes toward HPV among minors in Lebanon. To address this knowledge gap, we conducted a study that directly examined HPV awareness and vaccination rates among Lebanese secondary school students.

Our findings revealed that among the 406 participants, 126 (31.0%) of the students had high knowledge, similar to the percentage found among female university students (36.5%) [14]. Another study done in Turkey found that adolescents (25.28% females, 24.45% males) had poor knowledge of HPV compared to university students (62.46% females, 63.09% males) respectively [30], whereas in Brazil, 21.4% of adolescents showed adequate knowledge to HPV [31]. Moreover, our study found that 195 (48%) participants relied on the internet to access information on HPV and that 112 (27.6%) showed high conspiracy beliefs about vaccines. This can be explained by the fact that adolescents are relying more on social media to access information about HPV which can be beneficial [32], yet another study found that almost one-quarter of tweets shared during a 3-month interval period contained misinformation on the HPV vaccine, and these tweets were the most engaged compared to supportive posts [33]. Interestingly, another study conducted on United States citizens found that individuals getting their information from the internet had higher knowledge on HPV and its vaccine [34]. Moreover, a study conducted in Jordan during 2021 revealed a noteworthy correlation between belief in vaccine conspiracies and reluctance to receive the HPV vaccine [20].

The multivariable analysis found that having heard of HPV and having received sexual education outside of school compared to not, were significantly associated with high knowledge about HPV. These findings are good yet worrisome at the same time. Our results correlate with a study previously done in the United States, which also found that individuals who used the internet as a way to access information on HPV had higher knowledge scores [34], yet not all information on social media is correct [33]. Our educational system lacks proper sexual education, which explains why students who are getting sexual education outside of school are the ones who got higher scores on the HPV knowledge test.

Furthermore, our bivariate analysis showed that students who received prior sexual education either inside or outside the schools and those who had received at least one dose of the HPV vaccine exhibited a higher level of knowledge about HPV. A previous prospective study done in the United States of America found that students who were exposed to sexual education at school had higher knowledge on HPV compared to students who did not [35], and this can explain our finding concerning this matter. In addition, another study conducted in Brazil found that individuals who received the HPV vaccine demonstrated notably greater knowledge of HPV compared to individuals who did not receive the vaccine [36].

In addition, our results revealed that only 46 (11.3%) students had gotten at least one dose of the HPV vaccine, with significantly higher vaccination rate found among females compared to males (87.0% vs. 13.0%). A study carried out on Lebanese mothers in 2018 showed that the vaccination rate of their daughters against HPV was only 2.5% [12]. Vaccination rates in Lebanon remain low compared to other countries; in Brazil, the HPV vaccination rate among adolescents was found to be 48.9% [37], while it surpassed 80% in Australia [38]. It is worth noting that while Brazil and Australia have incorporated HPV vaccination into their routine immunization programs, Lebanon has not followed suit.

On the other hand, the discrepancy in the vaccination rates between men and women can be due to the lack of recommendations to vaccinate men in Lebanon. In fact, a previous study conducted on Lebanese physicians of different specialties found that they did not follow international guidelines in terms of recommending the HPV vaccine, with females being recommended the vaccine 6.8 times more than males [18]. Moreover, a previously done systematic review found similar discrepancies in the vaccination rates between men and women among both Canadian and US adolescents [39].

Additionally, we found that students with a lower mean household crowding index were significantly associated with higher HPV knowledge and that students whose fathers have a university level of education had low conspiracy beliefs. In contrast, a study done on adolescents in Brazil found no association between knowledge about HPV and sociodemographic characteristics [40]. Yet, according to a study conducted on female US citizens, disparities in knowledge about HPV were linked to sociodemographic factors [41]. In contrast, a study conducted in China found that students from high socioeconomic backgrounds had more HPV knowledge but were more concerned about vaccine efficiency and side effects compared to students from rural settings who were more accepting of the HPV vaccine [42].

On the other hand, it is important to highlight that 70 (17.3%) students had prior or are currently sexually active, with no significant difference in vaccination rate between those who have ever had a sexual activity compared to those who had not (28.3% vs. 16.0%). A research study carried out on college students in the United States revealed that sexually active students had a higher probability of being vaccinated against HPV in comparison to students who had not engaged in sexual activity [43]. Our results also emphasize the importance of the implementation of adequate sexual education in Lebanese high-schools, since 17.3% of our participants had, or are currently sexually active, which suggests that the current Lebanese generation is less conservative than the previous generations [14].

## Limitations

Our study has some limitations. First, the study design is cross-sectional, where data was collected using a questionnaire; hence, recall bias could not be avoided as well as over- or underestimation of questions. Second, selection bias is to consider because of the snowball sampling method used for the recruitment and since our questionnaire could only be filled if you had access to an internet connection and if you knew English. Henceforth, the results of our study cannot be generalized to the overall Lebanese population. In addition, the knowledge and conspiracy beliefs scales used in the questionnaire are not validated in the Lebanese population; however, the Cronbach’s alpha values were good for both scales (α = 0.89 for both), which make our results strong and decrease the importance of this limitation. Lastly, there is a possibility of residual confounding effect, since some variables were not included in our survey (such as the absence of information on prior STIs), which may have had an impact on our results.

## Conclusion

To our knowledge, this is the first study conducted in Lebanon that directly targets the minor population. Our study highlights the need for more comprehensive sexual education in Lebanese high schools, as well as the importance of spreading accurate information about HPV and its vaccine, since students are relying mainly on the internet to access information about HPV. The findings suggest that only a small percentage of Lebanese secondary school students have high knowledge about HPV, and that misconceptions and conspiracy beliefs about vaccines are prevalent. The study also sheds light on the need to promote HPV vaccination among males. Addressing these issues can improve public health and help prevent the spread of HPV and related diseases. Overall, this research can serve as a foundation for future studies aimed at improving HPV awareness and vaccination rates among Lebanese youth.

## Data Availability

All data generated or analyzed during this study are not publicly available due to the restrictions from the ethics committee. Reasonable requests can be addressed to the corresponding author.

## Abbreviations

HPV: Human Papillomavirus
HPV-KQ: Human Papillomavirus Knowledge Questionnaire
VCBS: Vaccine Conspiracy Belief Scale
CDC: Centers for Disease Control and Prevention
FDA: United States Food and Drug Administration
USD: United States Dollar
STI: Sexually transmitted infections
